# The *h*-Index: An Indicator of Research and Publication Output

**DOI:** 10.12669/pjms.39.2.7398

**Published:** 2023

**Authors:** Faaiz Ali Shah, Shaukat Ali Jawaid

**Affiliations:** 1 Faaiz Ali Shah, Associate Professor Orthopaedics, Lady Reading Hospital Peshawar, Pakistan; 2 Shaukat Ali Jawaid, Chief Editor, Pakistan Journal of Medical Sciences, Karachi, Pakistan

The analysis of research publications using statistical methods is called Bibliometry. There are many bibliometric indices to measure the research output of individual researcher.^1^ The *h*-index and impact factor(IF) are the most famous and widely used bibliometric indices. Jorge Eduardo Hirsch was Professor of Physics at the University of California who introduced the *h*-index (Hirsch Index) in 2005 for the first time. Hirsch defined *h-*index as “A scientist has index *h* if *h* of his or her *Np* papers have at least *h* citations each and the other(*Np-h*) papers have fewer than *h* citations each.” 2 As an example one author will have *h*-index-7 if he had published seven papers and each paper has been cited at least seven times by others.

The research performance of an individual researcher at micro level can be determined using *h*-index. Many commonly used databases such as Clarivate Analytics Web of science, Google Scholar and Elsevier’ Scopus automatically calculate *h*-index for their authors. Publish or Perish is a free software program that calculate h-index for authors who do not have a profile account in Google Scholar. The calculation of *h*-index may be different in different databases or resources because each database covers different journals and years of indexation meaning that the same author will not have same *h*-index value across all databases. 3

The *h-*index evaluates the cumulative scholarly impact of an author’s performance. It measures the quantitative(productivity) and qualitative(citations) research work of a researcher as a single number meaning that neither few papers which are highly cited nor too many papers with very few citations will produce a high *h*-index. Scientists with outstanding and highly cited papers on new discoveries or inventions but few in numbers can not have high h-index. For example, Albert Einstein has *h*-index of 4 or 5 despite being acknowledged globally as an outstanding Physicist. Harry Kroto is a Nobel laureate in chemistry who won the Nobel award with his single publication in 1985 despite his position at 264^th^ in global chemist list base upon *h*-index. Contrary Charles Darwin gained total citations of 77539, *h*-index of 680 and *i*-10 index of 331 because he was the author of many books which were cited by many researchers worldwide.^4^

There are several advantages of *h-*index. It is a reliable and robust indicator of scholarly achievement. It is applicable to researchers in individual capacity as well as to researchers groups, medical journals, publishers, projects, academic institutions, universities and even to countries. For example, the *h*-index of Pakistan Journal of Medical Sciences(PJMS) as calculated by Google Scholar is 36 with an impact factor of 2.340.^5^ The *h-*index can be used as a yardstick for comparing many researchers for fellowships in research or for grants application in the same field. It can also predict the future achievement of a researcher.^6^ H index is also becoming very popular in the science community and in the days to come it might become much more important to evaluate the scientific contributions of faculty members.^7^ Indexing agencies, it is said, also look at the hIndex of Editorial Board members while evaluating the standard of a biomedical journal.

It is simple to calculate. But what should be a good *h-*index? Hirsch^2^ was of the opinion that 20 *h*-index is Good,40 is Outstanding and 60 is Exceptional but after 20 years of research life. He further pointed out that approximately 84% of Physicists with Nobel Prizes had *h*-index of 30. The pattern of citation and publications are different across various disciplines of medical and health sciences. It is therefore very difficult to propose a competitive or acceptable *h*-index for recruitment and promotion of faculty or for funding and grants purposes. However, an h-index of Three and Five can be set as standard for assistant professor, 8 to 12 for associate professor and *h*-index of 15 to 20 is a good standard for appointment to full professor. In many disciplines a general rule is followed for an acceptable *h*-index with matching of numbers of years the author has been working in that discipline. In fact, Hirsch^2^ made an adjustment by combining *h*-index with author’s active research time to arrive, the *m*-index and is determined by h-index dividing on time since researcher’s initial publication. The *m-*index of One is Very good, Two is Outstanding and Three is Exceptional. The famous English physicist and author Stephen Hawking had an *m*-index of 1.6.

Some intrinsic limitations of *h*-index have been reported in the literature.^8^ The inability of *h-*index to consider the accurate position of a researcher in the author list of an article is the first limitation of *h*-index. The major and minor (or no) contributor in the research gained equal *h*-index. The second limitation of *h*-index is the influence of self citation of an author by quoting his or her earlier research publication with an intention to increase his *h*-index. The third limitation of *h*-index is the influence of researcher “scientific age or academic age.” Researchers with shorter scientific carriers may have less scientific papers and citations than with longer scientific life. Similarly, the female researches are particularly at disadvantage because of possible discontinuation of their research activities due to maternal or child bearing leaves. Fourthly selective publications or clinically less irrelevant but popular topics can increase *h*-index. Original articles may have less impact on *h-*index than review articles as the latter are more frequently cited. Lastly the worth or content of a research can not be taken into account while using *h*-index as citation matrix.

Due to the above limitations of *h-*index many complementary indexes or types of *h*-index have been postulated. These are grouped into two broad categories. The *h*-index, *g*-index, *h*(2) and m-quotient are designated as Productive Output Core as they describe the number of published papers. The second category include *m*-index, *hw*-index, *r*-index, *a*-index, and *ar*-index and they describe the Impact of research papers. Both groups although different but can complement each other.^9^ A study by Guraya et al has showed that some universities offer generous grants to researchers who have a high h-index and with more publications in leading well reputed journals that ensures more chances of its citations and elevation in the scientific rankings of the funding institutions. 10

The *h-*index has been extensively studied in many medical and surgical subspecialties including Orthopaedic surgery and positive associations have been documented between the *h*-index and academic ranks or positions and promotions.^11^-^12^ Atwan^13^ studied 567 faculty of Orthopaedics of Canada. Among the study participants 485(85.5%) were academic faculty and 82(14.5%) were clinical faculty. Individual *h-*index was obtained from Elsevier’s Scopus database. The median *h*-index of academic faculty was 8 while median *h-*index of clinical faculty was 2(p<0.001) Assistant professors had *h-*index of 4, Associate had 12 and full Professor had 28 *h-*index(p<0.05). The spine specialty had the highest *h-*index of 11(4.5 to 18.5) while foot and ankle had the lowest mean *h-*index of 3.5(2 to 7.5). Atwan concluded that Orthopaedic faculty of Canada has higher *h*-index than Orthopaedic faculty of USA.Varady^14^ determined the *h-*index of top 100 Orthoaedic surgeons of USA who were more active on twitter and noted that their mean *h-*index was 13.67±4.12. He concluded that social media influence was positively correlated with higher academic productivity as evident from higher *h*-index.

Currently there is not a single perfect bibliometric index which can accurately describe the impact of a researcher. Therefore, a combination of two or more metrics are advised.^15^ Many researchers have outstanding contributions to their field through their ideas, time, skills and mentoring. Kelly^16^ is of the opinion that it is distasteful to reduce the lifetime work of a researcher to a mere numerical value as Albert Einstein has rightly pointed out that: “Not everything that Counts is Countable, and Not Everything that is Countable Counts”.^17^
